# Assessment of the Main Natural Disturbances on Norwegian Forest Based on 20 Years of National Inventory

**DOI:** 10.1371/journal.pone.0161361

**Published:** 2016-08-29

**Authors:** Olalla Díaz-Yáñez, Blas Mola-Yudego, Rune Eriksen, José Ramón González-Olabarria

**Affiliations:** 1 School of Forest Sciences, University of Eastern Finland, Joensuu, Finland; 2 Norwegian Institute of Bioeconomy Research, Ås, Norway; 3 Forest Sciences Centre of Catalonia, Solsona, Spain; Ecole Pratique des Hautes Etudes, FRANCE

## Abstract

The re-measurement of permanent forest inventories offers a unique opportunity to assess the occurrence and impact of forest disturbances. The present study aims at exploring the main forest damages in Norway based on the extensive data of several consecutive national forest inventories during the period 1995–2014. Five of the most common disturbance agents in Norway are selected for analysis: *wind*, *snow*, *browsing*, *fungus* and *insect* damage. The analyses focuses on the frequency and variation along time, the average damage at stand level and the spatial patterns of damage occurrence, resulting in a characterization of the damage produced by disturbances in Norway. The highest damage occurrences by disturbance agent are due to browsing, snow and wind. Snow presents a decreasing temporally trend in damage frequency in the studied period. By forest type, mature and intermediate birch forest are found to be more affected by snow damage, whereas mature spruce forest is by wind damage. The results from this study provide support to the hypothesis that damages by autumnal moth (*Epirrita autumnata*) on birch are more common in mature stands. No major attacks from bark beetle (*Ips typographus*) are found, probably related to the lack of major storm damages in the period. Forest types susceptibility to fungus has no apparent variation over time except in the last years, as increased occurrence is observed on mature spruce stands probably correlated with warmer than average periods. Browsing damage causes the most severe losses, as expected, in young stands, and is allocated mainly on the most productive forests. Although some of the disturbances present locally moderate effects, the results show no major disturbances threatening Norwegian forests in the studied period. Finally, the Norwegian national forest inventory demonstrates its reliability as a basis to understand the occurrence and effects of major natural disturbances.

## 1. Introduction

Natural disturbances are a key factor in forest dynamics [1], and a reason for the potential distribution of terrestrial vegetation [2]. At the same time, disturbances are considered as a major threat to forest resources [3] and associated ecosystem services [4]. The impact either positive or negative, of natural disturbances greatly depends on their regime, and how the affected ecosystems are adapted to cope with the intensity and recurrence of those disturbances. High intensity (i.e. stand level or larger) events are usually attracting most of the attention of society and, arguably, the scientific community. Nevertheless, disturbances have an important impact on the evolution of a forest even when their intensity and associated severity are mild.

Understanding e.g. the establishment of a stand after a severe disturbance, or the diversification of species and structure coming from a frequent but mild disturbance, provides the forester with information that can be included in management plans and objectives. This assertion can be applied either, if the objective is to emulate through management the evolution pathways of a natural forest [5,6], account for expected losses when defining a management plan [7], or combine economic and risk mitigation objectives [8].

In this context, the occurrence, susceptibility and impact of natural disturbances on forest ecosystems has been studied at different temporal and spatial scales, relying on a variety of methodological approaches and data sources [4,9]. In general, disturbance regimes are defined by the frequency of the events, their spatial and temporal distribution and impact on forest [10,11]. Even if the components of a disturbance regime depend largely on the type of disturbance [12], the spatial and compositional characteristics of the forest also has an important role on modifying both the extent and severity of disturbances [13]. The variability on disturbance regimes presents a challenge when large areas and multiple disturbance types are to be analyzed, as the spatial and temporal frames should be large enough to reflect variations on the spatial patterns of the events recurrence. For instance, remote sensing tools have shown their usefulness to capture information on the spatial distribution and impact of different types of disturbances at the required spatial and temporal scales [14–16]. However, remote sensing tools rely on variations on the vegetation structure and vitality, being traditionally better adapted to capture large-scale, stand replacing forest disturbances [17]. In addition, they require from previous information about the type of disturbance causing the observed variation on forest conditions.

Field assessments on forest health, on the other hand, offer the possibility of identifying the disturbance agents, and provide reliable information about the rate of damage, even when it is small or located on non-dominant forest strata. This valuable information comes at a high economic cost if large areas are to be monitored over an extended period of time. National Forest Inventories (NFIs), although not designed for the specific purpose of assessing forest health, are considered a potential source of information that can produce and complement disturbance related information [18,19]. Although NFIs are not spatially continuous, they provide a good approximation at the national scale on the spatial distribution and temporal evolution of forest types and associated goods and services [20]. When measurements of forest health are included on the NFIs design, they become an excellent source of data for assessing the influence of forest characteristics on the occurrence and severity of natural disturbances. Examples on the use of NFIs for assessing occurrence and impact of disturbances can be found for pests or diseases [21–23], fire [24–27], wind and snow [28–31], or game related damage [32,33], among others.

The present study aims at exploring forest disturbances in Norway by their specific agent, with emphasis on *Snow*, *Wind*, *Browsing*, *Insect*, and *Fungus* related damage. The main focus is to identify spatial and temporal patterns on the occurrence of forest disturbances based on data from four consecutive measurements of the Norwegian NFI, entailing 1995–2014. Additionally, it provides an overall insight on the occurrence and impact of natural disturbances on the Norwegian forest, analysing the susceptibility of different forest types to be affected by disturbances, and an overall assessment of the damage levels.

## 2. Materials and Methods

### 2.1. Data sources

The data for the analysis was based on the Norwegian National Forest Inventory (NFI) collected during the 7^th^, 8^th^, 9^th^ and 10^th^ measurements, corresponding to the period 1995–2014. The Norwegian NFI is a systematic permanent inventory, where plots allocated on a 3x3 km grid are measured every 5 years. For each permanent plot, variables were measured at three levels: stand (1000 m^2^ around the plot center), plot and tree level (on a circular plot of 250 m^2^). Forest damage was measured at stand level. The plots included in the analysis were those with damage measurements available and located in *productive forest* (i.e. expected yield over 1 m^3^ ha^-1^ year^-1^ over bark [34]). During 1995–2005 damage was not recorded in plots that were in regeneration stage. For plots divided by a stand border (e.g. a plot has one part on forest and another on water, or on two forest types with very different productive parameters), only the forested part was considered or the first division when both parts were forested areas.

**Fig 1:**
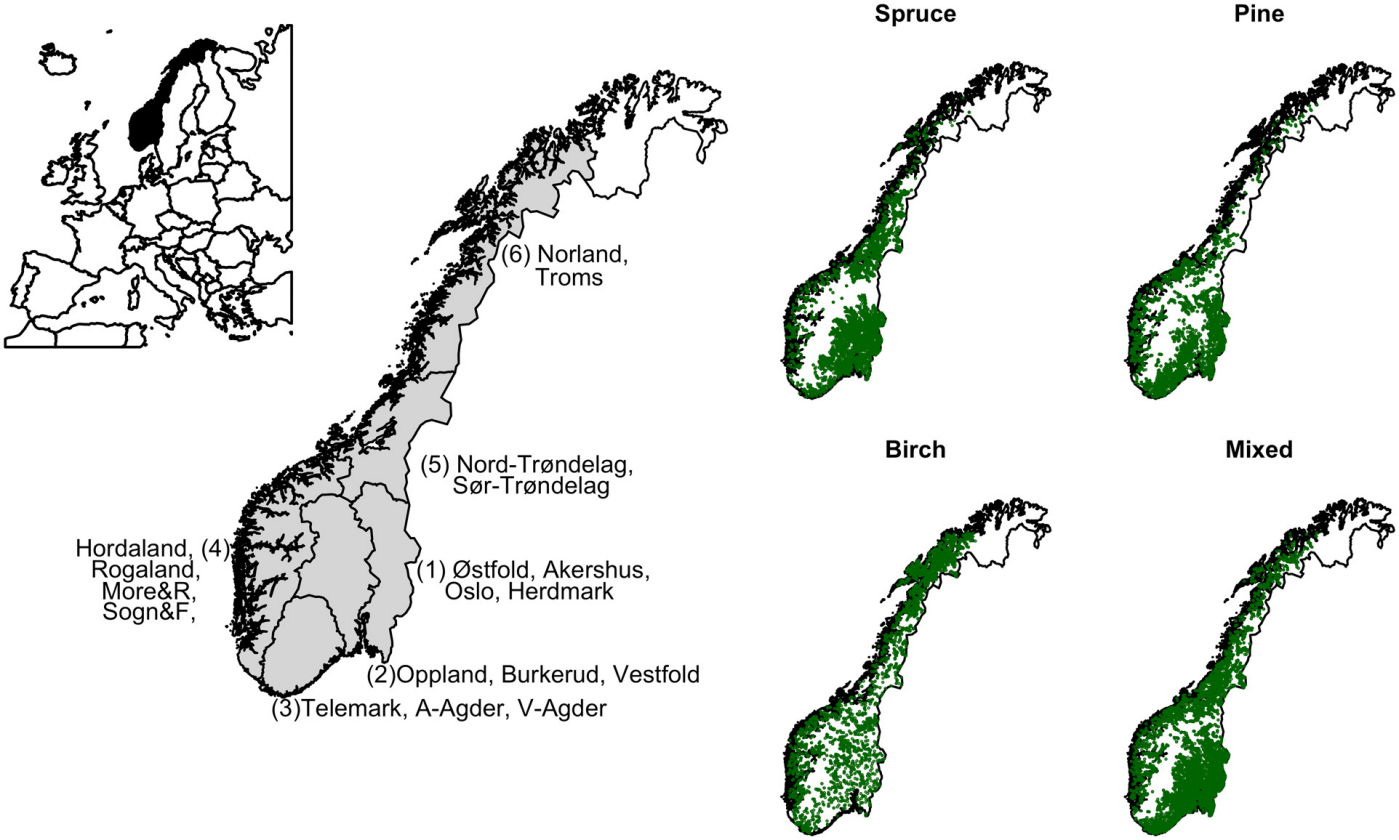
Studied area. Left: Studied area and regionalization used in the analysis. The counties were grouped in six main regions, except the northernmost county (Finnmark) that was not included in the analysis. Right: Spatial distribution of the national forest inventory plots by dominant species (spruce, pine, birch and mixed). Map border lines adapted from [35], original licensed under Creative Commons Attribution 4.0 International (CC BY 4.0).

In total 34 263 plot measurements were considered for the analysis, 8052, 8423, 8895, 8893 in the 7^th^, 8^th^, 9^th^ and 10^th^ NFI respectively; these plots entailing most of the forested parts of the country (Fig 1). The country was divided into regions as defined in the official statistics of forest condition and resources in Norway [34] (Fig 1). These regions are considered to have relatively similar forests, topography and climate (Table 1). The northernmost area of the country, Finnmark, was not included as the measurements in this county have started during the 9^th^ NFI with different grid in non boreal forest areas.

**Table 1:** Climatic characterization of the plots included in the analysis, by region.

Region	t_m_(°C)	t_min_(°C)	t_max_(°C)	P_sum_	N
**Region 1**	3	-0.7	6.7	664.7	8636
**Region 2**	2.4	-1.5	6.3	691.2	6490
**Region 3**	4.9	2	7.7	1049.6	5161
**Region 4**	5.5	2.8	8.1	1636.6	4347
**Region 5**	3.4	0	6.8	990.9	4889
**Region 6**	2.5	-0.5	5.5	1040.4	4740

Data based on the averages of the normal climatic period 1960–1990. t_m_: annual mean temperature t_min_: minimum temperature, t_max_: maximum temperature P_**sum**_: annual precipitation. N: Total number of plots included by region.

Following the NFI instructions, damage from different disturbance agents were recorded in each plot at each inventory following the criteria: it had an effect in the future economic development of the stand, it compromises the regeneration, or it represents a relevant decrease in the volume production or wood quality [34]. Damage was only recorded when it was detected to have occurred within 5 years prior to the NFI measurement.

In each plot, at least one disturbance agent was associated to the observed damage (Table 2). In the cases that several disturbance agents were reported at the same time in the same plot, the analysis only considered those ranked as the main disturbance agent (i.e. that occasioned the higher damage loss in the stand, as defined in [34]). Also at plot level, damage was quantitatively defined as a loss relative to volume, number of trees or crown loss according to the damage type and disturbance agent (Table 2) [34].

**Table 2:** Description of the disturbance agents and their associated damage as measured in the Norwegian National Forest Inventory.

Disturbance agent	Damage
Wind	Volume of trees blowdown by wind as a percentage of total volume.
Snow	Number of trees with snow break/blowdown in percentage of the total number of trees.
Drought	Volume of dead trees as a percentage of total volume.
Frost	Percentage of stand crown mass that is dead.
Fire	Volume of dead trees as a percentage of total volume.
Landslide	Number of trees with break / blowdown as a percentage of total number of trees.
Browsing	In old forest, it is the percentage of stand crown mass that is grazed away. In young forest it refers to the percentage of dead or damaged future trees as a percentage of the original number of trees.
Insects (includes: Bark beetle (*Ips typographus*), European pine sawfly (*Neodiprion sertifer*), Autumnal moth (*Epirrita autumnata*), Weevil (Curculionidae) and insect not specified)	Percentage of stands crown dead or fell off/ grazed away except for Bark beetle damage that corresponds with volume of dead trees as a percentage of total volume.
Fungus (includes: Spruce needle rust fungus (*Chrysomyxa abetis)*, Lophodermium needle cast pine (*Lophodermium)* and fungus not specified)	Percentage of stand crown that is dead or discoloured for Spruce needle rust fungus and other fungus no specified. And in young forest, percentage of dead future trees as a percentage of the original number of trees.
Mechanical (includes: Forest operations or damage by animal)	Volume of trees damaged in percentage of the total volume.
Mouse, beaver or ungulates	Volume of damaged trees as a percentage of total volume in old forest. For young forest it is the percentage of dead future trees from original number of trees.
Damage cause not known	Percentage of stands total crown that is dead.

The plots were divided according to their dominant species into four forest types. The criterion for assigning the forest type was the relative abundance of the main species expressed in volume distribution for older forest and crown cover for young forest. Therefore, plots were classified as spruce (spruce > 70%), pine (pine > 70%), birch (birch > 70%) and mixed forests (dominated by any or several of the previous species and with more than 10% of broadleaves). Plots were also divided according to their stand development class in three categories: young, intermediate and mature (corresponding with development class categories I and II, III and IV and V respectively in the Norwegian NFI [34]). The change from young to intermediate development class is mainly defined when the trees’ mean diameter exceeds 10 cm in spruce and pine forest, and 7–8 cm in birch forest.

### 2.2. Methodological approaches

Five of the most common disturbance agents in Norway were selected for further analysis: *wind*, *snow*, *browsing*, *fungus* and *insect*s. The analyses focused on the frequency of disturbance occurrence (presence of damage regardless of its impact), its temporal and spatial variation. The variation of the relative frequency of disturbance occurrence along the timeframe of the data was first visually explored by disturbance agent and region. In order to identify possible trends or peaks, linear and polynomial models were developed for each disturbance, identifying those with significant levels (p <0.05). The level of damage was also assessed quantitatively at plot level, and it was compared between regions.

The spatial analysis included the identification of areas with the highest concentration of plots affected by the occurrence of each of the disturbance agents. In this case, a geo-statistical approach based on kernel methods was used [36,37]. Kernel methods allow estimating the probability of occurrence of events in a continuous space. In general, the method requires to define a bandwidth parameter that will be use to aggregate the events. In our case, the events were defined as plots presenting damage by one of the disturbance agents selected. The approach taken was based on an adaptive bandwidth method [38] that varies the bandwidth size in relation with the estimated local amount of data (i.e. the size of the bandwidth is inversely related to the amount of data). Although fixed bandwidths are more common because of its simple and effective application [39,40]; it is widely known that they can present problems when dealing with populations showing high spatial inhomogeneity. A fixed large bandwidth will miss the finer variations of highly dense areas and a narrow one will increase the intensity function for isolated points. The global bandwidth needed to obtain the varying bandwidths was calculated based on the over-smoothing factor [41] and the pilot bandwidth was calculated with a leave-one-out least-squares cross-validation (following Bowman and Azzalini, 1997 [42]). Considering the large border effect that the Norwegian geography can have on the estimates, edge corrected adaptive densities were applied. For the Kernel calculations, the R package *sparr* [43] was used.

The frequencies of disturbance occurrence by development class of the forest (i.e. young stands, intermediate and mature) and dominant species were studied using contingency tables. The contingency tables were defined comparing the observed number of plots presenting damage by disturbance agent to the expected frequency values when the number of plots is divided proportionally between development classes. The difference was considered relevant when the observed value per cell was equal or larger than 1.5 the expected value (one-tailed), and otherwise it was assumed no effect of development class by species on the occurrence of the studied disturbance agent. Cells with large difference between observed and expected make a larger contribution to the Chi-squared test. The Chi-squared test was calculated against the null hypothesis that the different disturbance agents have the same effect on all forest types and development classes’ combinations (at the 0.05 level). The characteristics of the stand prior to the damage identification were based on the period before the damage was measured, for example if the disturbance occurrence was measured in the period 2000–2004 (8^th^ NFI) the stand characteristics before the damage corresponds with the information obtained during the period 1995–1999 (7^th^ NFI) in the same plot.

From the initial set of 34 263 plot measurements, all of them were used for temporal damage frequency analysis by disturbance agent. Relative frequencies of damage occurrence by disturbance were calculated relative to the number of plots measured in each year. The Norwegian NFI measures one fifth of all the plots in each year, therefore relative yearly estimations are possible to obtain. Damage was analyzed using the plots affected by one of the selected natural disturbances and with measurements of damage impact on the forest (2557 plots); these measurements cover the period (2000–2014) because prior to 2000 only information on disturbance occurrence (damage or undamaged for each plot) had been recorded. Finally, in the contingency analysis were also included plots with species composition and development class information before the damage (23 767 plots).

## 3. Results

Damage was detected in 3771 plots out of 34 263 studied plots during the period 1995–2014 (about 11%). In relation to the total plots available per forest type, birch forest was the most affected type, followed by mixed and spruce forests (17%, 10% and 8% of all plots, respectively). The highest disturbance occurrences corresponded to: browsing, snow and wind. By forest type, spruce forests were found to be more affected by snow, wind, fungus and browsing damage; pine forest was mainly affected by browsing, fungus and wind, birch forest by snow, insect attacks and browsing, and mixed forest by browsing, snow and wind.

**Fig 2:**
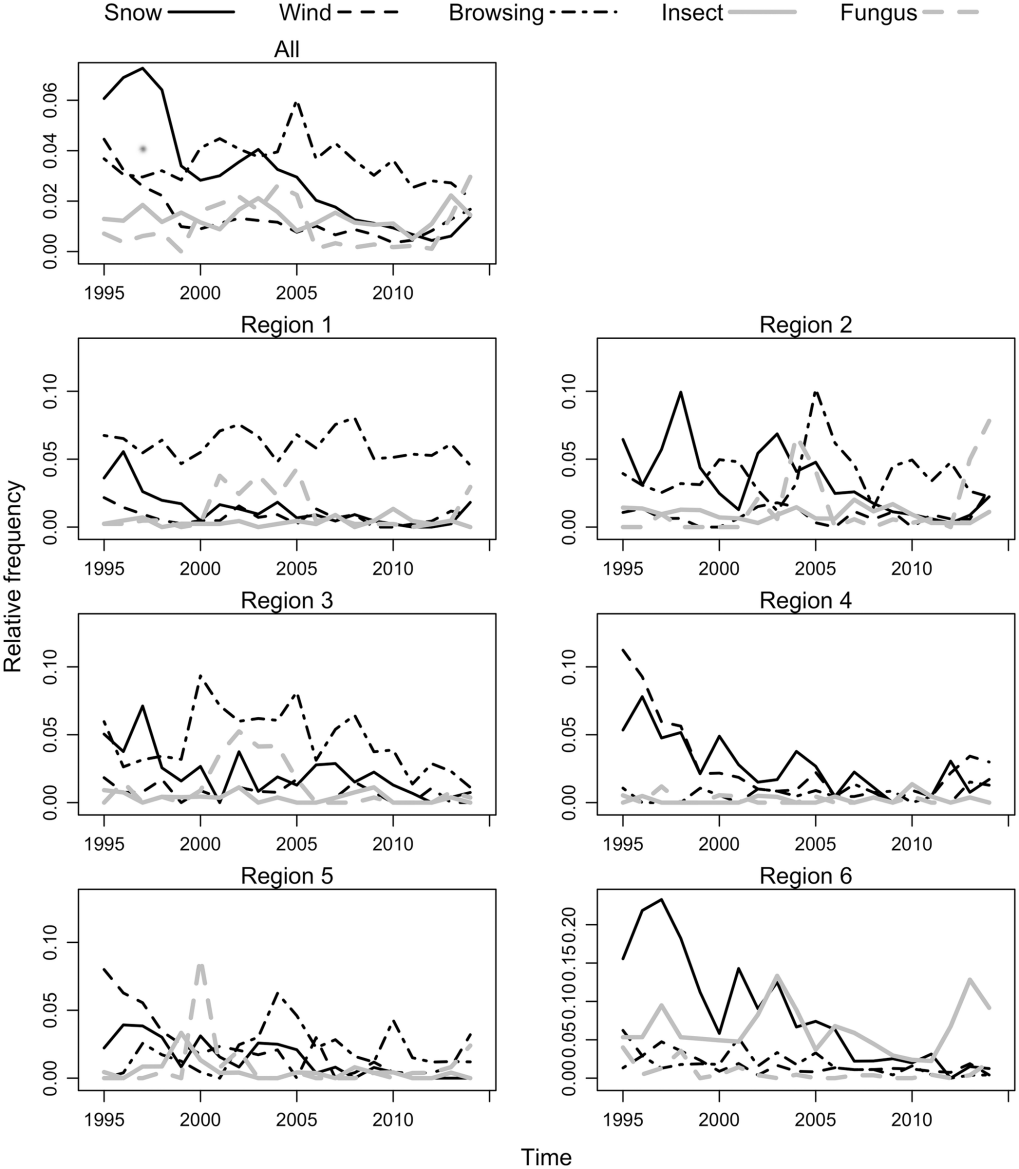
Frequencies of damage occurrence by disturbance agent along 1995–2014. Top: Relative to country level (*All*). Bottom: Relative to each region.

The time series showed some trends in the relative frequency of disturbance occurrences over time (Fig 2). At country level, the occurrence of snow and wind declined over time (linear trend, p<0.001 in both cases), although presenting a small increase in 2012–2014. Regionally, snow damage showed a similar trend in each of the 6 the regions (linear trend, p<0.001 for all regions), whereas wind showed a decreasing trend only in some regions (linear trend, p<0.001 only for regions 4, 5 and 6) as well as browsing (linear trend, p = 0.02 for region 6). Curves with peaks of frequencies were observed for browsing (significant for regions 3 and 5), and fungus (significant for region 6). In the case of snow and insect damage there were large occurrence pikes in region 6, corresponding to the years 1997, 2003 and 2013.

**Fig 3:**
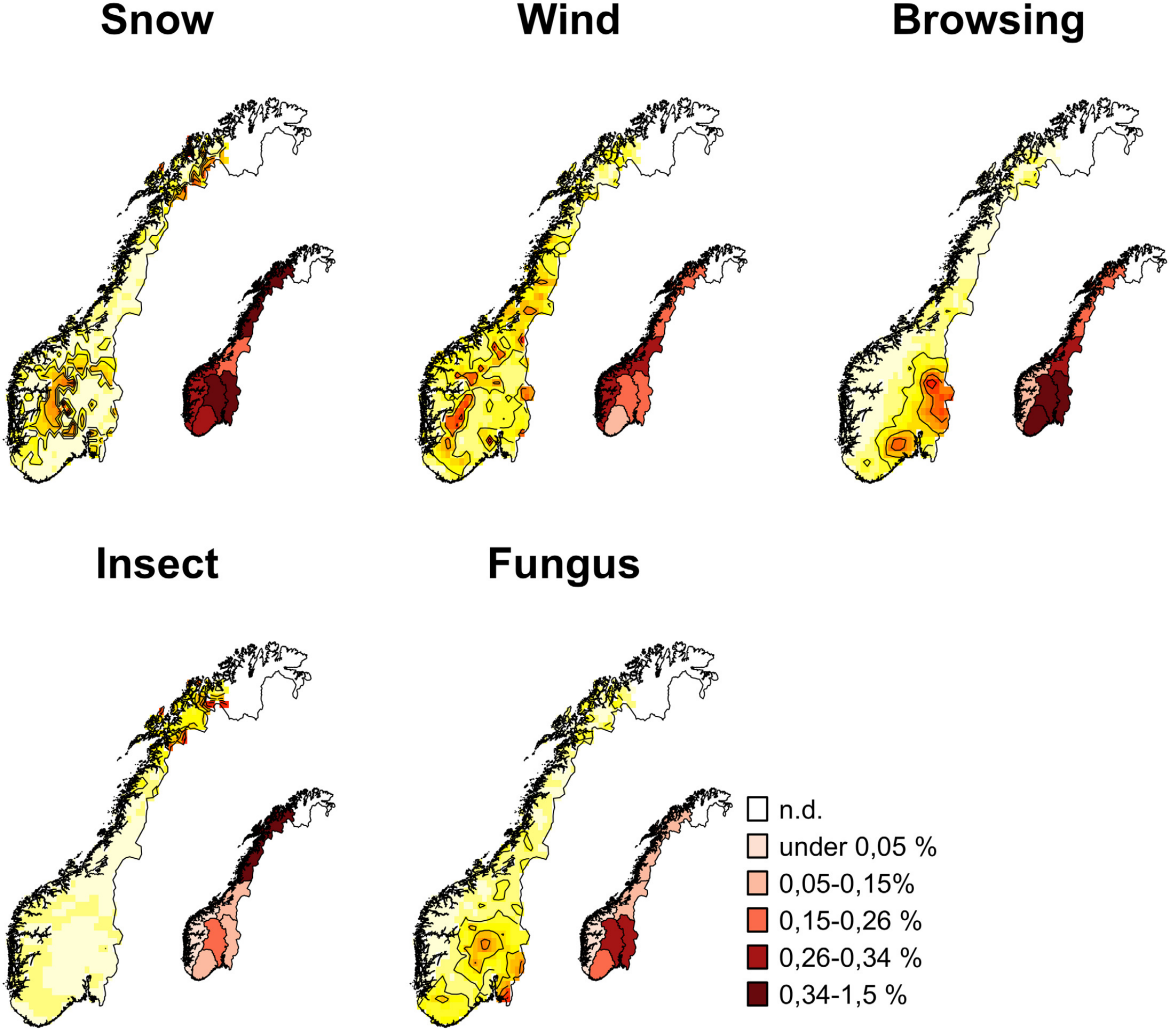
Damage occurrence spatial analysis. Spatial analysis of the locations with the highest concentration of damage occurrence (large maps) and average damage percentage by region (smaller maps) by disturbance agent during the period 1995–2014 (excluding *Finnmark*). For the large maps, darker colors represent higher concentrations of damaged plots, and the scale is relative for each map. For the small maps, the scale is defined in percentage of the total numbers of National Forest Inventory plots observed in the region. Map border lines adapted from [35], original licensed under Creative Commons Attribution 4.0 International (CC BY 4.0).

Each disturbance type showed a specific spatial distribution (Fig 3). Snow related damages were mainly located in the southern regions (region 1 and 2) and in the northern region (region 6), being specially clustered in mountainous areas. Wind related damage was more frequent on the western regions (region 4 and 5) and in mountainous areas facing the Atlantic side. Browsing damage was clearly aggregated in southeast regions (regions 1–3), forming two main hotspots. Insect related damage was most common in the northernmost of region 6, forming disperse hotspots. The occurrence of fungal damage was mainly present in the south of the country, with three main hotspots: in the coast, at the Swedish border and in the mountain areas of region 2.

It must be taken into account that damage due to insect and fungus responds to different fungal agents that are specific to a species, and the maps represent only the general occurrence of fungal damages altogether. Therefore, the contingency tables aimed at observing the damage by both species composition and development classes (Table 3), in order to identify difference occurrence rates for different species or for different development stages related to the stand’s age. The calculated Chi-squared was 4117.6 (d.f. = 55) with a p-value < 0.001, presenting a highly significant departure from the null hypothesis, meaning that the different damage agents affect differently to different forest types and development classes. For instance, the observed frequency for snow damage was considerably higher from expected values in mature and intermediate birch forests. The occurrence of wind damage was higher than expected in mature spruce forest (regions 2, 3 and 5). The occurrence of browsing damage was higher than expected in young stands of all forest types except in spruce stands. In spruce stands, all stages of development appeared to be less susceptible to browsing damage, except during the period 2005–09 where young spruce stands followed the same trend as other young forest types. Mature and intermediate birch forest were more susceptible to insect damage than other forest types. None of the forest types was found more susceptible of fungus damages over the whole study period. Although, during the last years spruce seemed to be more prone to suffer from fungus attacks. The effect of each of the disturbances agents, on the different forest types, was also tested showing that individually, the damage agents also affect differently to different forest types and development classes. The Chi-squared results were 458.46 for snow, 222.82 for wind, 2339 for browsing, 726.57 for insect and 70.44 for fungus, (d.f. = 11 and p-value < 0.001 for all cases).

**Table 3:** Contingency table presenting observed and calculated expected stand damage by disturbance agent.

		Observed/Predicted					
Development class	Spp. Composition	Snow	Wind	Browsing	Insect	Fungus	No damage
**Mature**	Spruce	4644	5522	1374	2829	3727	20512034
	Pine	549	1825	1184	333	3331	24472296
	Birch	13236	2518	661	13224	723	15411681
	Mixed	4440	3420	969	1627	1425	19471882
**Intermediate**	Spruce	6058	5329	1298	1039	3636	27812692
	Pine	342	1621	872	528	3726	20881967
	Birch	8227	614	846	8918	1117	11821257
	Mixed	6166	2633	16112	1344	3741	32313086
**Young**	Spruce	925	113	6943	017	2916	11821177
	Pine	210	05	10518	17	127	415488
	Birch	815	07	11225	810	99	606678
	Mixed	1452	026	42089	535	2933	22062439

Highlighted, those presenting a relevant deviation between observed and expected.

**Fig 4:**
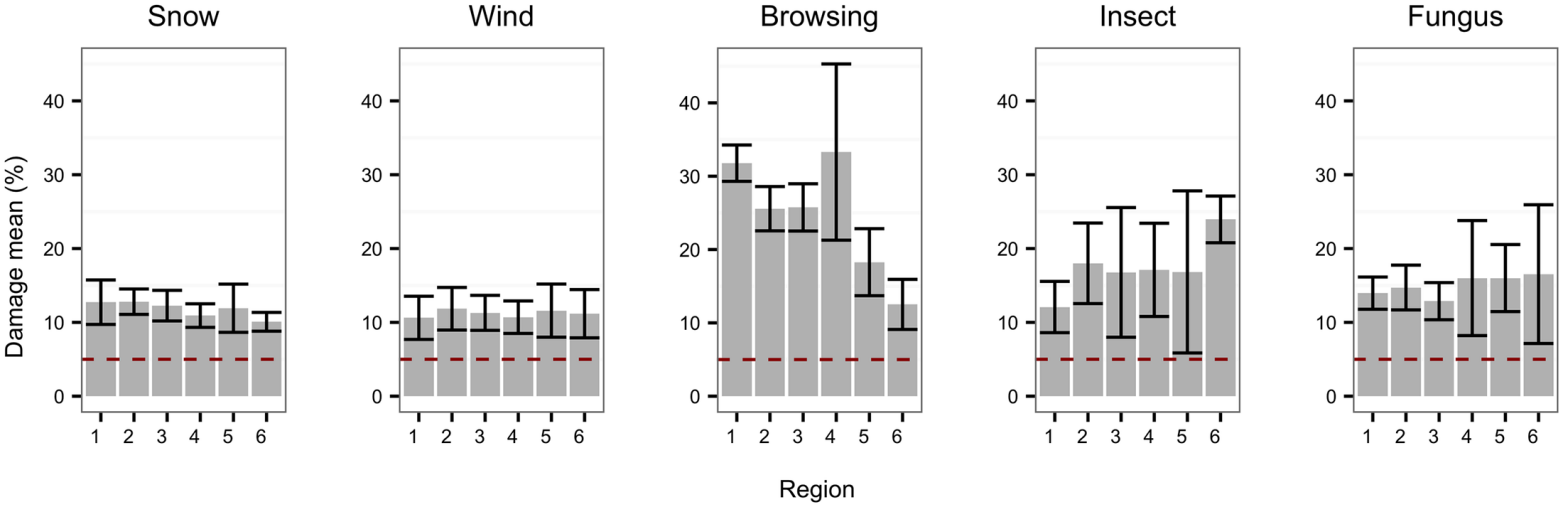
Average damage level observed per region (2000–2014) and disturbance agent. The dashed line represents the minimum damage value observed in the data (5%). The bands represent 2 x standard error associated to the mean. The damage is defined according to the National Forest Inventory instructions (see Table 2).

Finally, when the level of damage per affected plot was evaluated, it was found that the disturbance agent causing more severe losses was browsing, and especially on those plots located in regions 1–4 (Fig 4). The mean percentage of damage losses caused by snow, wind and fungus damages were not significantly different between regions. Most of the losses due to insect damage were in region 6 where the species composition is typically dominated by birch.

## 4. Discussion

Long-term damage analysis enhances the understanding of natural disturbances in forest areas [30] and it can help forecast the probability of damage given different timeframes. Previous studies have demonstrated the reliability of NFI data to assess the occurrence of the most important disturbances and their impact on forest resources [21,23,44], making NFI data a good basis for a characterization of disturbances in the forest. Variables from NFI have also been used to better understand forest factors related to occurrence or damage of different natural disturbances [24,28,32].

The present study evaluates the temporal evolution and spatial aggregation patterns of disturbance occurrence across Norway, based on several consecutive NFI datasets entailing all productive forest and showing its state and development (stock, species composition, health) over a 20 year period. The availability of four NFI measurements provides an excellent opportunity to evaluate similarities and divergences between disturbances, considering their temporal and spatial aggregation patterns.

When evaluating the evolution of disturbance occurrence, we observed clear variations between disturbance agents. The occurrence of snow and wind related damages appeared to peak in different years, but snow had a much higher peak between 1996–1997, and lower ones after this period. It was not possible to identify a clear fixed interval, which agrees with other European records where snow damaging events varied from each winter to longer cycles [45]. As expected, location seems to play a major role on defining the susceptibility to suffer from snow and wind damage [46]. As many other mountainous countries, Norway, presents a regionalized climate that can influence the spatial distribution of affected plots. Southern areas of the country are often influenced by southern weather systems whereas northern areas are influenced by oceanic clime, due to the proximity of the north Atlantic streams on the west. We found that the occurrence of snow and wind damages tended to be aggregated in mountainous areas, with no major differences along the north-south axis. Typically, northern trees are more adapted to this type of damage and also have a slower growth, allowing them to better adapt to wind and snow damage events [28].

When defining which forest types were more susceptible to be affected by wind and snow, our analysis showed that snow damage was more frequent on mature and intermediate birch forest, whereas wind affectation was associated to mature spruce, and both wind and snow damages were rare on pine forest. One factor that can explain this is the higher exposure of birch stands to the snow due to their location in mountainous areas. Another factor relates with structural parameters, e.g.: lower spacing between trees, compared to conifers. Our results partially disagree with previous studies indicating that snow and wind damage are more common in Scots pine and Norway spruce than birch [47,48]. However, for a similar diameter class, Scots pine would be more resistant to uprooting than spruce and birch stands, due to its deeper roots and better anchorage [47]. In addition to higher exposures due to location, one potential reason for the susceptibility of birch to snow damage could be that in Norway birch forest is usually shorter in height than spruce forest and their stems are easily bent or blow down by snow load or avalanches. Birch also has a tendency to regenerate through coppice in small groups of stems, generating gaps between regeneration groups that can ease the appearance of snow damage, for example due to avalanches [49]. In the case of spruce and wind damage, the higher susceptibility of the older stages of development was consistent with some studies [50] but disagreed with others [51]. It has to be mentioned that as the NFI data on wind damage focus on recording blown down trees, uprooting processes are overlooked in contrast to possible steam breakage, which is allocated as snow damage. These criteria in data recording can further explain our results, as larger specimens of spruce are more susceptible to uprooting [52] in contrast to smaller trees that often are more prone to break.

In the case of biotic agents, it is important to mention that the Norwegian NFI focused on some specific agents, both in case of insects and pathogens. For instance, in the case of insects, priority was given to bark beetle (*Ips typographus*), European pine sawfly (*Neodiprion sertifer*), autumnal moth (*Epirrita autumnata*) and weevil (*Curculionidae*), each of them typically specialized in certain tree species. The joined analysis of different and specialized insect and fungus agents had an important effect on all the dimensions of the occurrence analysis, and was therefore further explored by species in the contingency analysis. For example, insect damage was especially visible in a region dominated by birch (region 6), as most of the insect related damage corresponds to autumnal moth attacks on birch. The temporal evolution of the attacks of insect frequency showed cyclic occurrence peaks and agrees with previous studies where autumnal moth typically present cyclical outbreaks every 10 years [53,54]. Our results also provided support to the hypothesis that autumnal moth on birch is more common in older stands [55,56], as mature birch stands offer more favorable places for oviposition and an enhancement of food resources [55]. Another interesting result is the lack of major bark beetle outbreaks on spruce forest, during the period 1995–2014. This result is probably associated to the limited storm related damage for the same period and the well-known interaction between storm damage and *Ips* outbreaks [57–59]. The observed trend of limited but highly variable presence of bark beetle damage, agrees with the trends identified for the period 1972–2002 by Økland and Bjønstad (2003) [60], when no important outbreak was detected the years following the major attack of the 70s [61,62].

Concerning fungal attacks, the records also corresponded with different and specialised agents: spruce needle rust fungus (*Chrysomyxa abietis*) on spruce and lophodermium needle cast pine (*Lophodermium*) on pine. The selection of agents had a clear effect on the allocation patterns of the recorded attacks, as the most affected regions corresponded to those were spruce was the dominant tree species. Regarding the temporal evolution of the fungi attacks, they correlated with humid springs [63], and in many cases warmer than average periods such as the 2002–2004 and specially 2013–2014. When the susceptibility of forest types to fungi was analysed, no apparent variations were identified but during the last years analysed, where an increase on attack susceptibility was observed on young or mature spruce stands (S1 Table).

Browsing was one of the most relevant disturbance agents associated to Norwegian forests, both on occurrence and impact on the forest. Browsing frequency remained similar across time and the damage was allocated mainly on the most productive forests in the south of the country. Moose habitat selection is led by forage quality and shelter availability, both aspects changing in space and time [64]. Browsing affected mainly young stands, as typically moose prefer those because they offer more palatable forage at a reachable height [32]. The species composition of the stands also had an important effect on the allocation of browsing damage, due to specific food choices and preferences by ungulates [9,32]. Our study shows the preference by ungulates of mixed, birch and pine stands, except for the period 2005–2009 when spruce was also significantly damaged (S1 Table). Typically, moose prefer spruce stands the least [32], although it is also known that increased densities of moose population might also increase the risk of browsing damage (e.g. stands on migration routes or next to winter habitats). An important data limitation was that the 7^th^ and 8^th^ NFI (1995–2005) did not include damage on forests in regeneration stage. It would be reasonable to assume that the occurrence of browsing on those young stands would be even higher, as moose is known to browse in mixed stands with young pines (age<5 years) [65].

Despite the limitations, the Norwegian NFI has shown its potential as data source for assessing natural disturbances, their spatial and temporal distribution and the vulnerability of different forest types to them. We consider that the combination of four different NFI measurements provides a unique opportunity for this type of analysis. Our analysis suggests that a clear relation exists between wind and snow damage, which is supported by the recognized combined effect of wind intensity and snow load to induce storm related damage [28,51,66]. However, the instructions of the Norwegian NFI lead to consider stem breakage as snow derived damage (and never as a wind related damage) which could induce to misinterpretations. In this study we did not consider interactions among different disturbance agents due to the complexity of the analysis required and because of the absence of major abiotic disturbances that might trigger potential future damage by biotic agents [49,67–69]. The combination of different disturbance types within the same measurement was not considered, as the main disturbance was the only registered damage in over 85% of the damaged plots (S2 Table).

Finally, not finding major disturbances threating Norwegian forests is by itself the main result of the study. After the catastrophic storms and bark beetle attacks of the 70s [62,70], we may say that the Norwegian forest has sustained a relatively long period of good health, either for the absence of extreme storms, or due to the effects that past storms and biotic attacks had on the temporal depletion of susceptible trees [71]. This assumption calls for further analysis such as predictions of the forest evolution to detect potential threats in the future due to the natural ageing of the forest, or the inclusion of structural or management dependent variables, aiming at identifying past management actions that may help mitigate the impact of each disturbance. For example, the inclusion of more detailed information about snow loads and stand management history would also help define the importance of variations in the stand and trees’ structure on storm related damage.

The study of forest disturbances is an important source of information for developing a holistic management of forests and its associated ecosystem services [4]. The analysis of records of damage by different causing agents, with spatial approaches or with descriptors of forest stands can therefore help identify susceptible forest areas and at the same time, forest management practices that can reduce their probability.

## Supporting Information

S1 Table Contingency tables. Tables presenting observed (numerator) and the calculated expected (denominator) stand damage by development class and disturbance agent and Norwegian National Forest Inventory (NFI).
(DOCX)

S2 Table Disturbance agents simultaneously recorded in the same measurement. Percentage of the plots damaged by the main disturbance agent that were simultaneously damaged by a secondary and tertiary disturbance agent.
(DOCX)
