# Supplementary material for: Assessment of the Main Natural Disturbances on Norwegian Forest Based on 20 Years of National Inventory
Source: PLoS One. 2016 Aug 29;11(8):e0161361. doi: 10.1371/journal.pone.0161361 (PMC5003383; doi:10.1371/journal.pone.0161361)
Supplement: S1 Table — Tables presenting observed (numerator) and the calculated expected (denominator) stand damage by development class and disturbance agent and Norwegian National Forest Inventory (NFI). (DOCX) [file pone.0161361.s001.docx]

**S1 Table. Contingency tables and Chi-squared test results.**

**NFI 8 (2000-04):**

|  |  | **Observed/Predicted** | | | | | |
| --- | --- | --- | --- | --- | --- | --- | --- |
| **Development class** | **Spp. composition** | **Snow** | **Wind** | **Browsing** | **Insect** | **Fungus** | **No damage** |
| **Mature** | Spruce | 24/24 | 19/8 | 6/26 | 8/11 | 12/14 | 623/609 |
|  | Pine | 4/26 | 5/9 | 6/28 | 0/12 | 30/16 | 711/665 |
|  | Birch | 75/18 | 9/6 | 5/19 | 49/8 | 2/11 | 374/452 |
|  | Mixed | 18/23 | 17/8 | 4/25 | 7/10 | 8/13 | 601/576 |
| **Intermediate** | Spruce | 27/31 | 17/10 | 3/34 | 6/14 | 14/18 | 826/786 |
|  | Pine | 3/25 | 7/8 | 4/27 | 1/11 | 27/15 | 668/625 |
|  | Birch | 51/16 | 1/6 | 3/18 | 39/7 | 6/10 | 374/417 |
|  | Mixed | 36/40 | 14/13 | 6/43 | 3/18 | 21/24 | 1068/1010 |
| **Young** | Spruce | 7/15 | 0/5 | 22/16 | 0/7 | 9/9 | 392/378 |
|  | Pine | 2/6 | 0/2 | 39/6 | 1/3 | 7/3 | 119/148 |
|  | Birch | 7/8 | 0/3 | 34/9 | 3/4 | 7/5 | 191/213 |
|  | Mixed | 11/33 | 0/11 | 155/36 | 0/15 | 14/20 | 771/837 |

Chi-squared = 1665.6, d.f.=55, p-value < 0.001

**NFI 9 (2005-09):**

|  |  | **Observed/Predicted** | | | | | |
| --- | --- | --- | --- | --- | --- | --- | --- |
| **Development class** | **Spp. composition** | **Snow** | **Wind** | **Browsing** | **Insect** | **Fungus** | **No damage** |
| **Mature** | Spruce | 17/13 | 14/7 | 4/29 | 13/8 | 7/5 | 706/700 |
|  | Pine | 1/14 | 6/7 | 4/31 | 1/9 | 2/5 | 823/770 |
|  | Birch | 32/10 | 5/5 | 1/21 | 32/6 | 1/4 | 501/526 |
|  | Mixed | 14/11 | 10/6 | 5/25 | 3/7 | 2/4 | 640/620 |
| **Intermediate** | Spruce | 20/16 | 17/8 | 4/36 | 2/10 | 5/6 | 921/891 |
|  | Pine | 0/12 | 4/6 | 2/27 | 1/8 | 9/5 | 706/664 |
|  | Birch | 24/7 | 1/4 | 4/16 | 20/5 | 0/3 | 387/401 |
|  | Mixed | 19/18 | 9/9 | 4/40 | 5/11 | 12/7 | 1027/990 |
| **Young** | Spruce | 2/8 | 1/4 | 36/18 | 0/5 | 6/3 | 427/434 |
|  | Pine | 0/3 | 0/2 | 38/7 | 0/2 | 4/1 | 138/166 |
|  | Birch | 1/4 | 0/2 | 39/9 | 1/3 | 0/2 | 205/226 |
|  | Mixed | 2/15 | 0/7 | 152/33 | 4/9 | 3/6 | 706/798 |

Chi-squared = 1308.8, d.f.=55, p-value < 0.001

**NFI 10 (2010-14):**

|  |  | **Observed/Predicted** | | | | | |
| --- | --- | --- | --- | --- | --- | --- | --- |
| **Development class** | **Spp. composition** | **Snow** | **Wind** | **Browsing** | **Insect** | **Fungus** | **No damage** |
| **Mature** | Spruce | 5/6 | 22/7 | 3/20 | 7/10 | 18/8 | 722/726 |
|  | Pine | 0/8 | 7/9 | 1/23 | 2/12 | 1/9 | 913/863 |
|  | Birch | 25/6 | 11/7 | 0/19 | 51/10 | 4/8 | 666/707 |
|  | Mixed | 12/6 | 7/7 | 0/18 | 6/10 | 4/7 | 706/686 |
| **Intermediate** | Spruce | 13/9 | 19/10 | 5/27 | 2/15 | 17/11 | 1034/1018 |
|  | Pine | 0/6 | 5/7 | 2/18 | 3/10 | 1/7 | 714/677 |
|  | Birch | 7/4 | 4/4 | 1/12 | 30/6 | 5/5 | 421/437 |
|  | Mixed | 6/10 | 3/11 | 6/29 | 5/15 | 4/12 | 1136/1083 |
| **Young** | Spruce | 0/3 | 0/4 | 11/10 | 0/5 | 14/4 | 363/362 |
|  | Pine | 0/2 | 0/2 | 28/5 | 0/2 | 1/2 | 158/175 |
|  | Birch | 0/2 | 0/2 | 39/6 | 4/3 | 2/3 | 210/238 |
|  | Mixed | 1/7 | 0/8 | 113/21 | 1/11 | 12/9 | 729/799 |

Chi-squared = 1355.7, d.f.=55, p-value < 0.001

The NFI 7 it is not included as a table because we are presenting the characteristics of the stand prior to the damage identification, therefore for the disturbances occurred during 2000-2004 (8^th^ NFI) the stand characteristics before the damage corresponds with the information obtained during the period 1995-1999 (7^th^ NFI) in the same plot.
