# Supplementary material for: Assessment of the Main Natural Disturbances on Norwegian Forest Based on 20 Years of National Inventory
Source: PLoS One. 2016 Aug 29;11(8):e0161361. doi: 10.1371/journal.pone.0161361 (PMC5003383; doi:10.1371/journal.pone.0161361)
Supplement: S2 Table — Percentage of the plots damaged by the main disturbance agent that were simultaneously damaged by a secondary and tertiary disturbance agent. (DOCX) [file pone.0161361.s002.docx]

**S2 Table. Disturbance agents simultaneously recorded in the same measurement.**

|  | **Second damage** | | | | | | **Third damage** | | | | | |
| --- | --- | --- | --- | --- | --- | --- | --- | --- | --- | --- | --- | --- |
| **Main damage** | **No second damage** | **Snow** | **Wind** | **Browsing** | **Insect** | **Fungus** | **No third damage** | **Snow** | **Wind** | **Browsing** | **Insect** | **Fungus** |
| Snow | 85.6 | 0 | 2.1 | 1.6 | 5.2 | 3.5 | 99.8 | 0 | 0 | 0 | 0 | 0.2 |
| Wind | 89.2 | 3.6 | 0 | 0.4 | 2.4 | 1.6 | 96.9 | 0 | 0 | 0 | 0.4 | 0.4 |
| Browsing | 95.4 | 0.6 | 0.2 | 0.6 | 0.8 | 1 | 99.9 | 0.1 | 0 | 0 | 0 | 0 |
| Insect | 87.4 | 8.2 | 0.9 | 1.2 | 0.6 | 0.6 | 99.4 | 0.3 | 0.3 | 0 | 0 | 0 |
| Fungus | 92.9 | 4.2 | 0.3 | 1.3 | 0 | 0.3 | 100 | 0 | 0 | 0 | 0 | 0 |
